# Effect of electronic prescribing with formulary decision support on medication tier, copayments, and adherence

**DOI:** 10.1186/1472-6947-14-79

**Published:** 2014-08-28

**Authors:** Joshua M Pevnick, Ning Li, Steven M Asch, Cynthia A Jackevicius, Douglas S Bell

**Affiliations:** 1 Division of General Internal Medicine, Department of Medicine, Cedars-Sinai Health System, 8700 Beverly Blvd, PACT 400.8G, Los Angeles, CA 90048, USA; 2 UCLA David Geffen School of Medicine, Los Angeles, CA, USA; 3 Department of Biomathematics, UCLA David Geffen School of Medicine, Los Angeles, CA, USA; 4 Stanford School of Medicine, Palo Alto, CA, USA; 5 Veterans Affairs Palo Alto Health Care System, Palo Alto, CA, USA; 6 Institute for Clinical Evaluative Sciences, Toronto, Canada; 7 University Health Network, Toronto, Canada; 8 Department of Health Policy, Management and Evaluation, Faculty of Medicine, University of Toronto, Toronto, Canada; 9 Veterans Affairs Greater Los Angeles Healthcare System, Los Angeles, CA, USA; 10 Department of Pharmacy Practice and Administration, College of Pharmacy, Western University of Health Sciences, Pomona, CA, USA; 11 RAND Health, Santa Monica, CA, USA

**Keywords:** Clinical decision support, Electronic prescribing, Medication adherence

## Abstract

**Background:**

Medication non-adherence is prevalent. We assessed the effect of electronic prescribing (e-prescribing) with formulary decision support on preferred formulary tier usage, copayment, and concomitant adherence.

**Methods:**

We retrospectively analyzed 14,682 initial pharmaceutical claims for angiotensin receptor blocker and inhaled steroid medications among 14,410 patients of 2189 primary care physicians (PCPs) who were offered e-prescribing with formulary decision support, including 297 PCPs who adopted it. Formulary decision support was initially non-interruptive, such that formulary tier symbols were displayed adjacent to medication names. Subsequently, interruptive formulary decision support alerts also interrupted e-prescribing when preferred-tier alternatives were available. A difference in differences design was used to compare the pre-post differences in medication tier for each new prescription attributed to non-adopters, low user (<30% usage rate), and high user PCPs (>30% usage rate). Second, we modeled the effect of formulary tier on prescription copayment. Last, we modeled the effect of copayment on adherence (proportion of days covered) to each new medication.

**Results:**

Compared with non-adopters, high users of e-prescribing were more likely to prescribe preferred-tier medications (*vs.* non-preferred tier) when both non-interruptive and interruptive formulary decision support were in place (OR 1.9 [95% CI 1.0-3.4], p = 0.04), but no more likely to prescribe preferred-tier when only non-interruptive formulary decision support was in place (p = 0.90). Preferred-tier claims had only slightly lower mean monthly copayments than non-preferred tier claims (angiotensin receptor blocker: $10.60 versus $11.81, inhaled steroid: $14.86 versus $16.42, p < 0.0001). Medication possession ratio was 8% lower for each $1.00 increase in monthly copayment to the one quarter power (p < 0.0001). However, we detected no significant direct association between formulary decision support usage and adherence.

**Conclusion:**

Interruptive formulary decision support shifted prescribing toward preferred tiers, but these medications were only minimally less expensive in the studied patient population. In this context, formulary decision support did not significantly increase adherence. To impact cost-related non-adherence, formulary decision support will likely need to be paired with complementary drug benefit design. Formulary decision support should be studied further, with particular attention to its effect on adherence in the setting of different benefit designs.

## Background

Adherence to medications is a critical component of controlling chronic illness. Nonetheless, multiple investigators have documented adherence rates of approximately 60% [1], with little to no improvement over time [2]. Furthermore, meta-analysis shows this poor adherence to be associated with increased mortality [3]. Furthermore, the cost of adherence-related hospital admissions alone has been estimated at $100 billion annually in the US [4]. Our analysis focuses on the potential of electronic prescribing (e-prescribing) with formulary decision support (FDS) to help physicians know, and thus choose, the lowest-tiered medication within a given class. This choice could minimize patients’ copayments and thereby improve medication adherence.

Prior research has shown that FDS is associated with increased usage of generic and other lower-tiered medications [5-8]. Furthermore, lower copayments are known to be associated with improved adherence [9,10]. Evidence of these two relationships suggests that implementing e-prescribing with FDS could improve adherence. However, a 2014 issue brief found no direct study of this topic [11]. We sought to evaluate whether FDS could reduce patient medication costs, and thereby improve adherence.

## Methods

### Ethics statement

The RAND Corporation Institutional Review Board approved this analysis. The requirement for informed consent was waived because this was a retrospective analysis of existing health care data in which the researchers did not have access to identifiable patient information that would have allowed patients to be contacted.

### Study design

This was a retrospective difference in differences analysis of de-identified records from e-prescribing adopter and non-adopter cohorts before and after FDS implementation.

### Setting

In late 2004, Horizon Blue Cross Blue Shield of New Jersey (BCBSNJ) led an initiative to offer subsidized iScribe standalone electronic prescribing (e-prescribing) software to high volume prescribers. In a prior publication, we described levels of e-prescribing use among 297 primary care physicians (PCPs) who participated in this initiative by adopting iScribe during 2005 [12]. They were compared with 1892 PCPs who were also offered the e-prescribing system during this time period, but did not adopt it. We found that solo practitioners, pediatricians, and physicians with more patients from predominantly African American zip codes were less likely to adopt e-prescribing. In the current study, we compare the pharmaceutical claims (claims) of these PCPs’ assigned primary care patients before and after implementation of FDS.

### Isolating and classifying pharmaceutical claims

**Figure 1:**
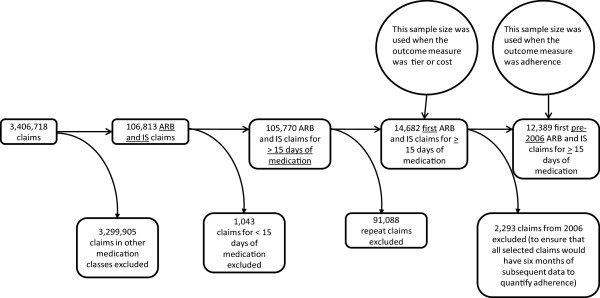
Pharmaceutical claim exclusion process.

We obtained a dataset containing all claims for medications dispensed between June 3, 2003 and July 21, 2006 and submitted to Horizon BCBSNJ for the assigned primary care patients of the 2189 PCPs. Because there are other ways of increasing brand to generic switches (e.g. state laws that require generic medications be dispensed when available), we selected two medication classes without generic medications available during the time period considered. We thus isolated all claims for angiotensin receptor blocker (ARB) and inhaled steroid (IS) medications (Figure 1). For the latter class, we recognize that adherence calculated from claims is often lower than for pills. Nonetheless, prior studies show that proportion of days covered (PDC) can be reliably measured within this lower range [13-16].

Then, we excluded claims with less than 15 days supplied to eliminate trial starts and a small number of claims that appeared erroneous. We further restricted the dataset to first, new claims (‘index’ claims) because the decision to select a given medication within a class is considered most when a medication is started. This restriction was accomplished by excluding any claims preceded by another same-class (ARB or IS) claim during the prior six months. Finally, for our adherence analysis, we also excluded index claims from 2006. Since all of the patients were continuously enrolled through June 30, 2006, this ensured that we had six further months of claims to calculate adherence subsequent to each index claim.

### Different pharmaceutical benefit plans, and their effect on patient copayments

Among studied patients, pharmaceutical benefit plan coverage was heterogeneous. The most common benefit plan used a three tiered formulary with ascending copayments for generic, preferred, and non-preferred brand medications. However, some patients had two tiered plans with identical copayments for all branded medications. Others had percentage coinsurance requirements that did not differ by brand status. A small proportion of patients were required to pay all costs at the pharmacy and later submit for reimbursement, in which case copayments could not be deduced from claims. Finally, even among patients with similar plan structures, there were differences in terms of actual copayment amounts, coinsurance percentages, deductibles, out-of-pocket maximums, flex spend plans, and “gap” insurance that would affect patients’ actual out-of-pocket costs. As with nearly every study using pharmaceutical claims as a data source, we did not have access to all of this cost information, but we nonetheless used the copayment amounts listed in the claims to model the overall relationship between copayment and tier. For example, if a patient’s plan required 10% coinsurance for a $200 claim, the patient responsibility on the claim would show as $20, so we would use $20 as the ‘copayment’ for that claim.

### Intervention – initial non-interruptive FDS changed to combined non-interruptive and interruptive FDS

**Figure 2:**
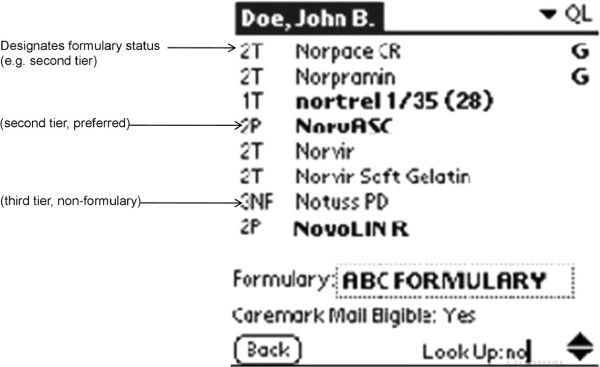
Passive formulary decision support indicates medication tier.

For the aforementioned e-prescribing initiative, Caremark began activating participating physicians’ e-prescribing software in January of 2005, and continued to do so on a rolling basis throughout 2005. We isolated the claims of patients attributed to the 297 PCPs studied in our prior manuscript, each of whom activated their iScribe e-prescribing software during calendar year 2005. For each study PCP, Caremark provided us with an e-prescribing activation date.During the study period, the software initially used only non-interruptive FDS, but later added interruptive FDS. Thus, e-prescribers were initially only exposed to non-interruptive FDS, which consisted of automatic display of the medication tier at the time of e-prescribing (Figure 2). Beginning September 16, 2005, the software exposed e-prescribers to both non-interruptive and interruptive FDS, which included the following: For the ARB medication class, physicians selecting candesartan, eprosartan, losartan, olmesartan, or telmisartan were advised to “Consider preferred brands Avapro, Diovan”. Atacand was added on February 17, 2006 after it also became a preferred brand. For the IS medication class, physicians prescribing inhaled beclomethasone, flunisolide, or triamcinolone were advised to “Consider preferred brands Flovent HFA, Pulmicort Turbuhaler”. Thus, for the purposes of our analysis, each study claim was linked to the appropriate PCP e-prescribing adoption date and subsequently classified as belonging to one of these three time periods: Pre-FDS, e-prescribing with non-interruptive FDS only, and e-prescribing with (both) interruptive and non-interruptive FDS.

Because there was no date of e-prescribing activation for non-participating PCPs, we assigned each non-participating PCP a ‘synthetic’ activationdate by random sampling with replacement from study PCPs’ actual activation dates. This assignment was done to make the distribution of activationdates similar in both groups, such that our analyses would be robust to secular trends. We then used these dates to separate control PCPs’ claims into pre and post-FDS claims. Because interruptive FDS was added on a specific date, we used this date to further classify post-FDS claims into the period of non-interruptive FDS only versus the period of both interruptive and non-interruptive FDS.

We could not definitively determine whether individual claims had been electronically prescribed, but we were able to associate each claim with a PCP’s level of e-prescribing usage. Because of our prior finding that levels of e-prescribing usage varied greatly but were generally stable, we classified e-prescribing users as high versus low users based on whether they used it more or less than 30% of the time. Based on our previous analysis of usage levels, this represented the 70^th^ percentile, and the mean usage rate among this subgroup was 61% (61 e-prescriptions per 100 claims) [12].

### Outcome variables – medication tier, patient copayment, and adherence

Medication tier was found in Horizon BCBSNJ formularies. Claims contained patient copayment data. Because cost variables often contain a skewed distribution with many outlying data points [17,18], a Box-Cox transformation was used to determine the best way of transforming the patient copayment variable to minimize root mean square error. Adherence was quantified using the proportion of days covered (PDC): after a patient filled a new medication, the PDC was the percentage of the subsequent 180 days when any medication within the index class (ARB or IS) was available to them, based on the days of medication supplied according to claims data [19].

### Covariates and intervention variables

Caremark provided physician specialty and practice size information. Horizon BCBSNJ provided de-identified demographics for each of the PCPs’ assigned primary care patients. As described in prior work, we used patients’ zip codes to estimate their household income, race (black vs white) and ethnicity (Hispanic vs non-Hispanic) [12]. Dosing frequency was calculated from claims data.

### Data analysis

We first compared characteristics of the three groups of studied PCPs and their patients, including across the three time periods studied. We made bivariate comparisons between FDS use and medication tier, between tier and patient copayments, between patient copayments and adherence, and finally directly between FDS use and adherence. We then constructed four multiple regression models to control for possible confounders.

Because our prior work found that usage of the FDS intervention varied widely [12], the FDS:tier model includes the interaction between the extent of FDS usage and the type of FDS present. We used claims from non-participating PCPs in corresponding times periods (before e-prescribing activation, after activation of e-prescribing with non-interruptive FDS, and after the addition of interruptive FDS) to control for secular trends. A difference in differences approach was used to compare the temporal differences within like PCPs across groups of PCPs. The estimated effects in this model were obtained from generalized estimating equations (GEEs) with a logit link function.

The tier:copayment model used multiple linear regression, and assumed that insurers only consider tier and medication class in determining copayments. These covariates were therefore the only ones tested. Linear mixed effects models were used to examine copayment:adherence and FDS:adherence associations. In the copayment:adherence and FDS: adherence regression models, a one-dimensional random effect was used to control for clustering of patients within PCPs.

Because these three underlying models required irreconcilable specification differences, the final model that directly analyzed the relationship between FDS and adherence was not just an identical, overarching model, but rather a separate analysis. The regression models were generally constructed by beginning with all available and theoretically tenable predictor variables included, and then using a backward variable selection procedure to eliminate covariates determined not to be associated. A p-value threshold of 0.05 and model fit criteria were jointly used to make this determination. Model fit was assessed using the quasi-likelihood information criterion for the FDS:Tier model and the Akaike's information criterion for the copayment:adherence model. All analyses were performed using SAS, release 9.2 (SAS Institute, Inc; Cary, NC).

After developing these three models, we used the FDS:tier and tier:copayment model estimates to project the effect of FDS on patient copayments. We also used the tier:copayment and copayment:adherence model estimates to project the effect of tier on adherence, and we combined all three model estimates to project the effect of FDS on adherence. Finally, because the FDS:tier model was the most important new knowledge generated in our analyses, and because there is extensive prior evidence regarding tier:copayment and copayment:adherence relationships, we combined our FDS:tier model estimates with this prior evidence. Specifically, we used annual survey results from the Kaiser Family Foundation that included copayments for different medication tiers to summarize existing knowledge of tier:copayment relationships, and we use a landmark meta-analysis of cost-sharing studies to understand copayment:adherence relationships [20,21]. In doing so, we generated FDS:copayment and FDS:adherence illustrative projections that were independent of the tier:copayment and copayment:adherence relationships we found in the studied setting. Adherence projections assumed a baseline PDC of 60%.

## Results

**Figure 3:**
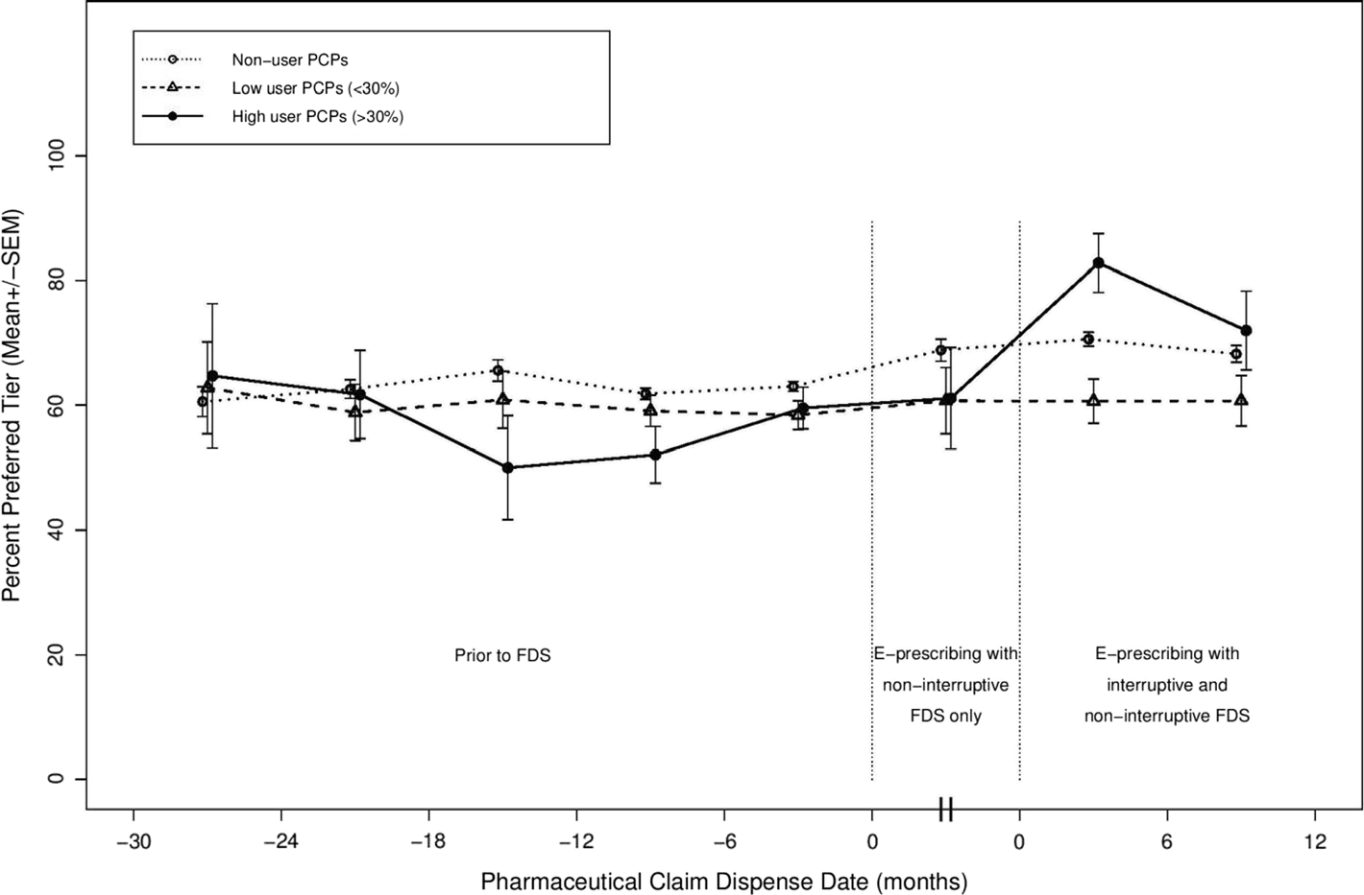
Unadjusted percent preferred medication tier in each of three user groups over time*.

Table 1 summarizes differences between PCPs, patients, and claims included in the analyses. A similar comparison across the three studied time periods found them to be generally similar, except that more patients of pediatrician PCPs had index claims in the latter two time periods. Our prior publication included 2189 PCPs [12], but only 1831 PCPs had patients with index claims that triggered inclusion here. Figure 1 shows how many claims were excluded in each of the steps described above. The bivariate comparison of FDS use and tier shows that high user PCPs exposed to interruptive and non-interruptive FDS selected preferred brands more often than low and non-users, and more often than high users with only non-interruptive FDS (Table 2). Within any one group of PCPs, the largest measured change in preferred tier prescribing occurred with the addition of interruptive FDS among high user PCPs, as their preferred tier prescribing increased to 78%. This represents a significant increase from the prior 61% preferred tier prescribing among these PCPs when using non-interruptive FDS only. In that time period, this group’s rate of 61% was not different from other PCPs. Because we found a significant secular trend of increasing preferred brand usage across the three time periods, even among the non-user PCPs, we continued to use synthetic adoption dates in the regression analyses to account for this secular trend.Before developing a model, we graphed the unadjusted proportion of claims for the preferred medication tier in each of three user groups over time (Figure 3). This figure shows that the groups were statistically similar in the pre-FDS period and remained so when non-interrruptive FDS was added, but that the high user group had a higher proportion of preferred tier claims after interruptive FDS was added. This increase appeared to moderate in the final measurement period. When we constructed two separate figures for ARB and IS claims, the patterns over time were similar, but IS claims were more likely to be preferred tier.

**Table 1:** Characteristics of physicians, patients, and pharmaceutical claims included in the analysis

**Characteristics**		**Non-users**	**Low users**	**High users**	p-value*
**PCPs**					
N (%)		1570 (86%)	187 (10%)	74 (4%)	
Specialty					
	Family practice	490 (31%)	67 (36%)	28 (38%)	0.03
	Internal medicine	838 (54%)	103 (55%)	35 (47%)	
	Non-surgical	2 (0.13%)	1 (0.53%)	1 (1.4%)	
	Pediatrics	235 (15%)	16 (8.6%)	10 (14%)	
Practice size					
	1 physician	783 (50%)	64 (34%)	29 (39%)	<0.01
	2–5 physicians	612 (39%)	90 (48%)	39 (53%)	
	6–10 physicians	148 (10%)	30 (16%)	6 (8.1%)	
	11–25 physicians	20 (1.3%)	3 (1.6%)	0 (0%)	
	> 25 physicians	2 (0.13%)	0 (0%)	0 (0%)	
**Patients (PCPs’ primary care patients)**					
N (%)		12327 (86%)	1505 (10%)	578 (4.0%)	
Age, mean ± SD		45.8 ± 19.5	49.2 ± 16.0	47.7 ± 18.3	<0.0001
Female, N (%)		6367 (52%)	776 (52%)	285 (49%)	0.55
Neighborhood income					
	<45 k	2132 (18%)	261 (18%)	92 (17%)	0.13
	45 k – 75 k	6970 (58%)	878 (60%)	310 (56%)	
	> = 75 k	2899 (24%)	321 (22%)	152 (27%)	
**Claims (pre-2006 ARB and IS index claims for >15 days of medication)**					
N (%)		12563 (86%)	1533 (10%)	586 (4.0%)	
ARB claims, N (%)		9094 (72%)	1238 (81%)	448 (77%)	
Frequency > once daily, N (%)		1563 (12%)	125 (8.2%)	65 (11%)	
**E-prescribing adoption dates**		(Synthetic)			
Median		August 4, 2005	July 27, 2005	August 19, 2005	0.60
1^st^ quartile		June 2, 2005	June 2, 2005	June 8, 2005	
3^rd^ quartile		September 29, 2005	September 27, 2005	October 12, 2005	

*Fisher’s exact test was used for categorical comparisons because of low or zero counts in some cells. ANOVA was used for continuous variables.

**Patients having an index ARB or IS claim.

**Table 2:** Unadjusted percent preferred medication tier in each of three user groups in each of three time periods

**User Group/Time period**	**Prior to FDS**	**E-prescribing with non-interruptive FDS only**	**E-prescribing with interruptive and non-interruptive FDS**	p-value*
	Percent Preferred Tier				
Non user PCPs	63%	69%	70%	<0.0001	
Low user PCPs (<30%)	59%	60%	61%	0.87	
High user PCPs (>30%)	57%	61%	78%	<0.001	
All PCPs	62%	67%	69%	<0.001	
p-value	<0.01	0.19	<0.001		

*p-values are for comparisons within user groups across time and within each time period across user groups.

The multiple regression results in Table 3 demonstrate that patients whose PCPs were high users in a time period with both types of FDS were more likely to have preferred tier claims, and this association persisted even after adjusting for medication class (OR 1.9, p = 0.04). Because medication class itself had an unexpectedly strong effect in our model, we also constructed separate models for each class and found the odds ratios to be similar (ARB OR 2.0, p = 0.02; IS OR 1.6, p = 0.27). The statistical significance of the latter model was affected by the lower number of claims in this model, which only represented 27% of the claims in the joint model. We used parameters from the joint model to estimate that the net effect of interruptive alerts were 15% and 8% increases in the probability of preferred tier prescribing for ARB and IS, respectively.

**Table 3:** Logistic regression evaluating the relationship between formulary decision support and preferred medication tier (n = 14660)^*^

**PCP or Claim characteristic**^**†**^	**Odds ratio**	**95% CIs**	**P value**
Medication class – inhaled steroid	4.1	3.4-5.0	<0.0001
E-prescribing with formulary decision support (FDS) usage			
Low users (<30% of time)	0.9	0.8-1.1	0.35
High users (>30% of time)	0.8	0.6-1.1	0.16
Time periods			
Non-interruptive FDS time period	1.1	0.9-1.3	0.42
Interruptive and Non-interruptive FDS time period	1.0	0.9-1.1	0.59
Interactions			
Low users during time period with Non-interruptive FDS only	0.9	0.6-1.5	0.74
Low users during time period with Interruptive and Non-interruptive FDS	0.8	0.6-1.1	0.13
High users during time period with Non-interruptive FDS only	0.9	0.4-2.3	0.83
High users during time period with Interruptive and Non-interruptive FDS^‡^	1.9	1.0-3.4	0.04

^*^Variables without association, and therefore not used as predictors in this model, are: date dispensed, PCP prescribing volume with this insurer, patients’ average pharmaceutical claims per month, and patient race and income (as estimated from zip code data).

^†^The referent categories were as follows: angiotensin receptor blocker medication class, non-users, and claims from the time period prior to e-prescribing activation.

^‡^As noted in the text, this odds ratio remained in the range of 1.6-2.0 in five other models where we: (1) excluded pediatricians completely, (2) controlled for physician specialty, (3) controlled for practice size, (4) restricted to ARB only, and (5) restricted to IS only.

A Box-Cox transformation found that the monthly copayment variable was best represented in the tier:copayment and copayment:adherence regressions as the monthly copayment to the one fourth power. This transformation made the regressions more robust to outlying data points that commonly cause a right-skewed distribution of cost data. Compared to non-preferred brands, preferred brand ARB and IS medications had estimated median monthly copayments of $10.60 and $14.86, versus $11.81 ($1.21 higher) and $16.42 ($1.56 higher) for non-preferred brands, respectively (p < 0.0001). Given the aforementioned odds ratio of 1.9 and its associated 15% and 8% expected increases in the probability of using preferred tier medications, high users of e-prescribing with both interruptive and non-interruptive FDS would be expected to decrease patients’ median copayments for ARB and IS medications by $0.18 and $0.12 per month on average, compared to non-users of FDS (for ARB, $0.18 = $11.81 – [15% × $10.60 + (100% – 15%) × $11.81]) ).

When we substituted the tier:copayment relationship present in our dataset with national survey data from 2013 [21], we projected that the studied FDS would have reduce expected medication copayments by $3.49 (based on increasing the likelihood of receiving a preferred brand prescription with a $29 copayment by 15%, and decreasing the likelihood of receiving a non-preferred brand prescription with a $52 copayment by 15%). This is much greater than the expected lower monthly copayments of $0.12 - $0.18 associated with FDS calculated from our observed tier:copayment relationship.

Bivariate comparisons of adherence showed that lower copayments were associated with higher adherence rates. Multiple regression demonstrated that these associations were still present even after adjustments were made for medication class, dosing frequency, and patient income (Table 4). Using a log transformation of copayment did not substantially alter the estimates or p values in this model.

**Table 4:** **Linear mixed effects regression model evaluating the relationship between monthly copayment and medication adherence (n = 12389**^)*^

**Patient or Claim Characteristic**^**†**^	**Adherence, expressed as absolute percent change in proportion of days covered**	**P value**
$$\sqrt[{4}]{\mathrm{Copayment}\;\mathrm{in}\;\mathrm{dollars}}$$	-8%	<0.0001
Medication class – inhaled steroid	-49%	<0.0001
Zip code-based estimates of patients’ annual income^‡^		
$45 k – $75 k	6%	<0.0001
> $75 k	8%	<0.0001
Medication dosing frequency greater than once daily	-4%	<0.0001

^*^Variables without association, and therefore not used as predictors in this model, are: patient age, gender, history of depression (as evidenced by prior use of antidepressants), and patient race (as estimated from zip code data).

^†^The referent categories were as follows: angiotensin receptor blocker medication class, annual income < $45 k annually, and medication dosing less than or equal to once daily.

^‡^Estimated based on zip code data. 336 claims were excluded from this analysis due to missing zip code data.

We projected that the aforementioned copayment differences of $1.21 and $1.56 associated with the use of a preferred brand medication would be expected to raise PDC by 0.40 percent (ARB: 74.62% to 75.02%, IS: 73.34% to 73.74%). However, the aforementioned lower copayment of $0.18 associated with high use of e-prescribing ARB with both interruptive and non-interruptive FDS would only be expected to increase PDC by 0.06 percent. (Using $11.18 as an initial expected ARB copayment, -0.08 * [(11.00)^0.25^ – (11.18)^0.25^] = 0.06%). Although we projected this estimate by linking three underlying regression estimates, there was not a statistically significant direct association between high FDS usage and medication adherence, using either bivariate comparisons or multiple regression.

We also combined prior estimates of tier:copayment and copayment:adherence associations with our FDS:tier model to project the effect of FDS on adherence in a manner robust to the tier:copayment and copayment:adherence relationships of our studied setting. We thus applied our estimate of the effect of FDS on tier to the aforementioned survey data [21] regarding copayment differences and to a metanalysis of the copayment: adherence relationship [20]. Using these estimates, we projected that a patient of a PCP who frequently used e-prescribing with both interruptive and non-interruptive FDS could expect to realize cost savings that would translate to an increase in PDC of 3.6%.

We calculated this number using the expected cost method explained above, with the previously cited copayments of $29 and $52. Using the 15% increase in preferred tier prescribing, this yielded an expected copayment decrease of $3.49 associated with high usage of FDS. Using the midrange value of the Goldman et al. [20] estimate of copayment:adherence associations (a 4% decrease in out of pocket spending associated with a 10% increase in cost sharing), and assuming an initial PDC of 60%, we determined that high usage of FDS could be expected to increase PDC by 3.6%.

## Discussion

We found significant associations between high usage of interruptive FDS and medication tier, tier and copayment, and copayment and adherence within a single population of physicians and patients. However, predominantly because of modest copayment differences between tiers, there was no significant direct relationship between FDS and adherence. We begin by comparing each of the initial three estimates with prior findings.

Because no prior studies concentrated on the effect of FDS in medication classes without generic alternatives, it was difficult to directly compare our estimate of the association of FDS with medication tier to those found in these studies [6,8,22]. Given that there are other methods of encouraging generic substitution that are now widely used, we would hold that our study is a much more important test of the ability of FDS to reduce patients’ drug costs. Indeed, generic substitution has been successfully encouraged with broadly targeted educational campaigns and incentives [23]. In contrast, encouraging the use of preferred tier medications requires current and patient-specific formulary data not easily accessed without FDS.

To be sure, although we detected a relationship between *high* usage of FDS and medication tier, this association became non-significant when all FDS users were considered. A separate study of the effect of e-prescribing on generic prescribing also reported low usage rates (20%, versus 23% in our findings), and adjusted for these low rates to show the effect of FDS [22]. Greater e-prescribing usage will be needed for FDS to make an overall impact on medication tier or adherence.

We did find that high usage of e-prescribing with interruptive and non-interruptive FDS was associated with nearly 2-fold increased odds for prescribing preferred tier medications. Such an effect on preferred tier selection has not been demonstrated before. In today’s environment of increasing FDS usage, this finding should encourage pharmacy benefit management companies to apply increased tier price differentials and thereby enhance their power to negotiate lower pharmaceutical prices, with greater confidence that prescribers will select preferred medications. In turn, as tier price differentials increase, observers will be better able to assess whether, and to what extent, FDS may be used to increase adherence. One caveat to implementing FDS in today’s clinical environments, which may be already overflowing with sundry forms of clinical decision support, is that clinicians may be more prone to alert fatigue [24] than the studied PCPs. Indeed, these PCPs may have adopted standalone e-prescribing partially because they lacked EHRs.

Unlike the combination of interruptive and non-interruptive FDS, the non-interruptive FDS alone showed no improvement in preferred brand prescribing. We attribute this in part to the somewhat cryptic symbols shown in Figure 2, and recommend clear, intuitive interfaces [25].

The claims we studied showed a very weak tier:copayment association. Indeed, we estimated that preferred brand ARBs and ISs cost patients only $1.21 and $1.56 less per month than non-preferred brands, respectively. In contrast, the aforementioned survey data showed average preferred brand copayments of $29 and non-preferred brand copayments of $52 in 2013 (even in 2005, during the study time period, this survey showed copayments of $23 and $40, respectively), versus mean monthly copayments of $22.95 and $26.61 for these tiers in our data set [21]. Although this survey data did not specify the days of medication supplied, the copayment difference between tiers was more than ten times greater. The weaker association between tier and copayment we found was likely at least partially because the actual claims data we used from various pharmaceutical benefit plans in our dataset differed from the preferred/non-preferred data points gathered in the survey (various examples of plans leading to situations where the ‘preferred’ brand might not have had a lower copayment are given in the *Pharmaceutical Benefit Plans* section above). Despite these differences in the types of data obtained, the much weaker tier:copayment association we measured shows that the drug benefit plans we studied use less cost sharing than most US plans.

This weak tier:copayment association likely limited the effect of the FDS intervention on adherence in two ways. First, physicians would be more likely to disregard FDS once they learned that the copayment difference between tiers was minimal. Second, the limited ability of these recommendations to substantially reduce copayments would have tempered their effect on cost-related nonadherence. For these reasons, we believe this ‘weak link’ in the hypothesized overarching relationship between FDS and adherence to be the main explanation for an undetected significant relationship between these two variables.

We also compared our model of the effect of medication copayment on adherence with prior findings. Goldman et al. systematically reviewed prior literature and found each 10% increase in cost sharing was associated with a 2% to 6% decrease in out of pocket spending, which should usually correlate with adherence, at least as measured by claims [20]. We tested our model with several average monthly copayments ranging from $5 to $25 to determine that a 10% increase in cost was associated with a 0.3% to 0.4% decrease in PDC. Thus, our measured effect size was approximately one order of magnitude smaller than prior estimates. We found no obvious cause for this discrepancy.

Given that our tier:copayment and copayment:adherence associations were much weaker than prior findings and may have been unique to the studied setting, we also combined prior estimates of these relationships with our FDS:tier model to predict the most likely effect of FDS on adherence. We applied our estimate of the effect of FDS on tier to the aforementioned survey data regarding copayment differences and to the Goldman et al. estimates of the copayment:adherence relationship. Using these estimates, we projected that a patient with a PCP who used e-prescribing with both interruptive and non-interruptive FDS more than 30% of the time could expect to realize cost savings that would translate to an increase in PDC of 3.6 percent.

Our results suggest that FDS can only be expected to substantially impact adherence among patient populations whose PCPs are predominantly high users of FDS. Indeed, the observed changes in adherence were substantially limited by low physician usage of FDS. “High” user PCPs used FDS as infrequently as 30% of the time, and had mean usage rates of 61% [12]. However, since the time of our study, multiple government incentives [26,27] have increased nationwide e-prescribing usage rates to 44% of all prescriptions dispensed [28]. Thus, a substantial proportion of today’s highest FDS users are likely realizing PDC gains above 3.6%. Today’s higher copayment differentials would also increase the effect of FDS on adherence.

Beyond confirming the three hypothesized relationships, we found several other results to be consistent with prior findings. For example, higher dosing frequency and lower patient income were associated with decreased adherence. Our estimated adherence rates for ARBs were vastly greater than those for IS medications, which is consistent with prior reports [9,13-16]. At least some of this difference may be attributed to seasonal prescribing of IS for allergic asthma. We have thus also considered that our measured usage of preferred tier IS medications could underestimate true use of preferred tier IS among new users, at least to the extent that seasonal users requesting previously successful, but non-preferred tier IS medications could have been erroneously identified as new users in our analysis, because we only looked back six months to identify new users. Nonetheless, we found much lower rates of preferred tier use IS users than among ARB users. This may have been due to greater differences (perceived or real) among ARB versus IS medications, or it could also have stemmed from the proportion of preferred tier medications available (two preferred tier ARB out of seven total ARB, versus two preferred IS out of only five total IS). We found similar tier:copayment relationships in each class, suggesting that copayment was not a major factor explaining differential prescribing of preferred tier medications across classes.

There were also novel findings. Most notably, we initially found an unexpectedly strong association between pediatric PCPs and use of preferred brand medications (OR 11.2, p < .0001). Although including this predictor did not alter the OR of 1.9 for e-prescribing with interruptive and non-interruptive FDS among high users, we decided not to include this predictor in the FDS:tier model described in Table 3. It was excluded because there were only a small number of claims from patients with pediatric PCPs, and because this small number of claims substantially affected the overall FDS:tier model such that the predicted probability of preferred tier medications differed substantially from our raw data.

## Limitations

One limitation of our analysis was that we could not determine whether individual claims had been electronically prescribed. Usage rates were instead estimated at the physician level from a 2006 usage evaluation period, and our prior work showed that usage was relatively stable [12]. Lacking a prescription-level linkage probably biases our estimates toward the null hypothesis, but given the mean usage level of 61% in the high-use group it is also reasonable to view our estimates as more similar to the real-world effects one might find from an effective e-prescribing program. We also lacked data on the potential use of e-prescribing by control physicians (obtained outside of the studied program that offered e-prescribing with FDS to all of the included physicians), but survey data even more recent than these claims showed only 4% of US physicians had adopted “fully functional” electronic medical records that included e-prescribing capabilities [29]. Only a subset of this 4% would have had FDS, and a smaller subset would have been using FDS frequently. Thus, we believe that unmeasured usage of FDS did not substantially bias our results. If there were bias, it would be towards the null hypothesis.

Even though we examined data from just one standalone e-prescribing product implemented in 2005, we expect our results and conclusions to generalize, and to be included in updates to a recent issue brief that found no evidence on this topic [11]. E-prescribing products used today have more features, improved usability, and are more likely to be part of an integrated EHR. Although these conveniences are surely helpful, they use the same formulary and benefits standard to transmit the same information via the same interruptive and non-interruptive alerting mechanisms we studied. To be sure, the early adopting physicians who began using e-prescribing in 2005 could differ from physicians who have not yet adopted e-prescribing today, and we cannot exclude the possibility that these differences could lead to a different result from the use of FDS.

We focused on the benefit of FDS in medication classes without generic options. Although many commonly used medications, including ARB and IS, now have generic alternatives, a variety of other mechanisms are being used successfully to increase generic substitution. Furthermore, new drug classes are continuously emerging, with generics naturally lagging, and formulary tier will continue to be used as a tool for competitive contracting.

To best address these issues, we considered many representative candidate classes. The ARB and IS classes were the best choices in terms of containing clinically similar, multi-tiered medications without generic alternatives during the three year study time period. We acknowledge that there are subtle clinical differences between the medications in these classes. Nonetheless, these differences need not restrict the choice of initial medication, and our difference in differences design means that any PCP perceptions regarding superiority would have been very unlikely to affect our results. Finally, although we believe that the characteristics of these medication classes are similar to many other classes used for chronic diseases, it would be important to study whether our findings generalize to acute care, where e-prescribing may be less prevalent.

Another limitation is that we used a difference in differences methodology to analyze the results of a real world initiative, rather than a planned experiment. Because PCPs themselves decided whether they would adopt and use FDS, there may have been selection bias. For example, PCPs more attuned to copayments may have been more likely to adopt and use FDS. However, high users’ unadjusted preferred tier usage of 57% before e-prescribing was the lowest of any PCP group in any time period, suggesting that these users were not predisposed to choosing preferred brands. Nonetheless, if the high user group possessed some other characteristic (e.g. more educated patient populations, who might both be healthier and have less aversion to lower-tiered medications) that made them more amenable to low tiered prescribing when exposed to the intervention, then FDS might not work the same for other physicians lacking this characteristic. This limitation is common to nearly all observational studies.

As with most any study, more data could have improved our analysis. For the time period with e-prescribing and interruptive FDS, upon which we relied for our most important results, we had over nine months of data as a result of a one-time data extract for a larger project [30]. Nonetheless, more claims would be useful to observe whether, and to what extent, the observed increase in preferred tier prescribing persisted over time.

Our transformation of the copayment variable has the advantages of improving model fit and satisfying the assumptions behind the statistical model. However, it can hamper interpretability, and partially for this reason we have provided several examples of how copayment differences are associated with differences in tier and adherence. Finally, we studied a patient population with a heterogeneous mix of pharmaceutical benefit plans, which provides good generalizability because it resembles real-world conditions. As in most cases, this generalizability comes at a cost of reduced internal validity. To the extent that studied plans deviated from a tiered benefit plan with set copayments, our results would have been biased towards the null hypotheses. Internal validity might be further optimized, though external validity adversely impacted, by studying PCPs whose patients had identical pharmaceutical benefit plans.

## Conclusion

In evaluating the relationship between FDS and adherence, we have provided the most direct evidence to date that FDS can be useful in helping physicians to choose preferred brands, rather than just contributing to generic substitution. In the studied population, interruptive FDS shifted prescribing toward preferred tier medications, but these medications were only minimally less expensive for patients. Thus, FDS did not significantly increase adherence. To impact cost-related non-adherence, FDS will likely need to be paired with complementary drug benefit design. Combining our estimate of the FDS effect on tier with more generalized prior findings regarding tier:copayment and copayment:adherence relationships demonstrates that FDS holds promise for policymakers and health system leaders to increase adherence.

## Competing interests

This data was initially gathered during work on grant 1U18HS016391-01 funded by the Agency for Healthcare Research and Quality as part of a larger set of e-prescribing pilot studies (Dr. Bell). This work was also supported by a Clinical Scholars Research Grant from the Burns and Allen Research Institute at Cedars-Sinai Medical Center (Dr. Pevnick), and by the National Center for Advancing Translational Science, Grant UL1TR000124 (Drs. Bell and Pevnick). The authors have no conflicts of interest to disclose.

## Authors’ contributions

JP and DB contributed to study concept and design, data acquisition, data analysis and interpretation, drafting of the manuscript, manuscript revisions, and statistical analysis. NL contributed to data analysis and interpretation, drafting of the manuscript, manuscript revisions, and statistical analysis. SA and CJ contributed to drafting of the manuscript and manuscript revisions. All authors read and approved the final manuscript.

## Pre-publication history

The pre-publication history for this paper can be accessed here:

http://www.biomedcentral.com/1472-6947/14/79/prepub
